# Survival rate and marginal bone loss in titanium vs titanium-zirconia single-unit narrow diameter implants. A systematic review and Meta-analysis of prospective studies

**DOI:** 10.4317/medoral.27042

**Published:** 2025-04-06

**Authors:** Juan Francisco Peña-Cardelles, Samuel Akhondi, Jovana Markovic, Kevser Pala, Alejandro Lanis, German O Gallucci

**Affiliations:** 1 Department of Restorative Dentistry and Biomaterials Sciences, Harvard School of Dental Medicine, Boston, Massachusetts

## Abstract

**Background:**

Implant-supported single-unit restorations present a predicTable and widely used treatment option to replace missing teeth. Narrow-diameter implants provide a viable treatment option for clinical scenarios with limited bone availability, minimizing the need for bone augmentation procedures. This systematic review aims to assess implant survival and implant marginal bone loss among different single-unit narrow implant diameters and between titanium single-unit and titanium-zirconia single-unit narrow implants.

**Material and Methods:**

This article was structured according to the Preferred Reporting Items for Systematic Reviews and Meta-Analyses (PRISMA®) Included criteria were randomized clinical trials (RCTs) and clinical prospective studies with at least 10 patients, a mean follow-up period of at least 12 months, and assessing single-tooth implants with a diameter of 3.5 mm or less.

**Results:**

Twelve included articles were prospective clinical studies, while 3 articles were randomized controlled clinical studies. There are no significant differences in the comparison between the 2.9-3.0 mm group and the 3.3-3.5 mm group (*p*=0.933). Both groups had a 99% survival (95% CI, 99.9%-100%). Titanium-zirconia implants presented a mean of marginal bone loss of -0,37 mm (CI, 95%: -0,53 to -0,20) meanwhile, titanium implants had a mean of marginal bone loss of -0,43 mm (CI95%:-0,71 to -0,28).

**Conclusions:**

The findings reveal no significant differences between 2.9-3.3mm and 3.3-3.5mm groups within narrow implants. Both titanium and titanium-zirconia alloys in narrow implants, exhibit comparable high survival rates and minimal marginal bone loss, showcasing their feasibility as a viable treatment alternative in scenarios with limited space or inadequate bone for regular diameter implants.

** Key words:**Implant survival, marginal bone loss, titanium implants, titanium-zirconia implants, narrow diameter implants, single-unit implants.

## Introduction

Implant-supported single-unit restorations present a predicTable and widely used treatment option to replace missing teeth (1,2). Certain clinical scenarios can pose a challenge for the use of regular diameter implants due to limited bone availability and/or mesiodistal space (3-5). Narrow-diameter implants offer a treatment alternative for these clinical situations since they require less bone and space than regular diameter implants (6,7).

A consensus report proposed the following classification for narrow diameter implants: category 1 (diameter <2.5 mm), category 2 (diameter 2.5 mm to <3.3 mm), category 3 (diameter 3.3 mm to 3.5 mm) (8). It was stated that implants with a diameter less than 2.5 mm demonstrated significantly lower survival rates than regular diameter implants, whereas implants with a diameter of 2.5 mm and more showed no statistically significant difference in survival rates compared to regular diameter implants (8).

Narrow-diameter implants can reduce the need for bone augmentation procedures since they need less native bone to be surrounded by. Moreover, the invasiveness of the surgical procedure minimizes trauma to surrounding tissues (9,10). Therefore, the use of narrow-diameter implants can shorten the treatment time and decrease patient morbidity compared to regular diameter implants which can require additional bone grafting procedures. Narrow implants also tend to have more simplified and efficient osteotomy protocols compared to regular diameter implants. Furthermore, in cases with a narrow emergence as maxillary lateral incisors, narrow diameter implants can allow for a more natural-looking emergence profile (7,11). Disadvantages of narrow-diameter implants may include an increased fracture risk and/or mechanical complication due to the thin material thickness at implant-abutment connection, presenting the need for more long-term data on the clinical performance (9). Nevertheless, studies have shown reliable results in the anterior and premolar regions (12).

This systematic review aims to assess the implant survival and implant marginal bone loss among different single-unit narrow implant diameters and between titanium single-unit and titanium-zirconia single-unit narrow implants.

## Material and Methods

This systematic review was organized according to the Preferred Reporting Items for Systematic Reviews and Meta-Analyses (PRISMA) guidelines (13). A focus question was developed using the Population, Intervention, Comparison, and Outcome (PICO) framework:

"In patients receiving single-crown implant-supported restorations (P), does the use of 2.9mm-3.0mm (I) diameter implants compared to 3.3mm-3.5mm (I) diameter implants (C) and different materials (I) (titanium and titanium-zirconia) (C) demonstrate differences in implant survival rates and marginal bone loss (O)? “

Included criteria were randomized clinical trials (RCTs) and clinical prospective studies with at least 10 patients, a minimum age of 18 years at the time of implant placement, a mean follow-up period of at least 12 months, and assessing single-tooth implants with a diameter of 3.5 mm or less.

- Information sources and search strategy

A comprehensive literature search until August 2023 was conducted in the MEDLINE (via PubMed), Science Direct, Scopus, the Web of Science and Cochrane Library databases. A search for nonpeer-reviewed studies was conducted on the OpenGrey database. Additionally, the references cited in the chosen articles were reviewed to identify any publications that were not found in the initial search but could be pertinent.

The search was performed by 2 independent researchers (J.F.P-C.,S.A). MeSH (Medical Subject Headings) terms, keywords and other free terms were used with Boolean operators (OR, AND) to combine searches: (dental implant AND narrow diameter OR small diameter dental implants OR "narrow‐diameter dental implants" OR "narrow dental implants" OR "small dental implants" OR "mini‐implants") AND (survival OR success).

- Study records

Two researchers (J.F.P-C. and S.A.) independently compared results to enhance completeness and eliminate duplicates. Following this, the titles and abstracts of the remaining articles were individually screened. Ultimately, full-text articles were chosen for this systematic review based on the specified criteria. Any disagreements were resolved through discussion with a third reviewer (G.G.), reaching a consensus. The agreement between reviewers was assessed using the Kappa coefficient and expressed as a percentage of concordance. If needed, the study authors were contacted for clarification or to obtain missing information.

Data extraction included the following study characteristics: authors, publication year, study design, sample size (patients and implants), type of intervention, follow-up duration in months, implant failures, implant survival, complications, implant length, loading type, tooth replaced, presence of guided bone regeneration, and marginal bone loss. These data were verified by two independent researchers.

The extracted data was recorded using Microsoft Excel Software and any discrepancies or uncertainties in the data extraction process will be solved by third independent research.

- Meta-analysis

A meta-analysis was conducted to evaluate the variables of success, survival, and complications using Stata Release 15 software (StataCorp LLC). For the meta-analysis of survival data, the fixed effects method was applied because the studies showed homogeneity as indicated by the Cochran Q statistic

- Risk of bias in individual studies

The authors evaluated the quality and risk of bias of the included studies using the Newcastle-Ottawa Scale. This assessment was performed independently and in duplicate by two authors (J.M. and J.-F.P.-C.). Any disagreements were discussed between them, and a third researcher was consulted if a consensus could not be reached. Refer to Table 1 for details.

## Results

- Study selection

**Figure 1:**
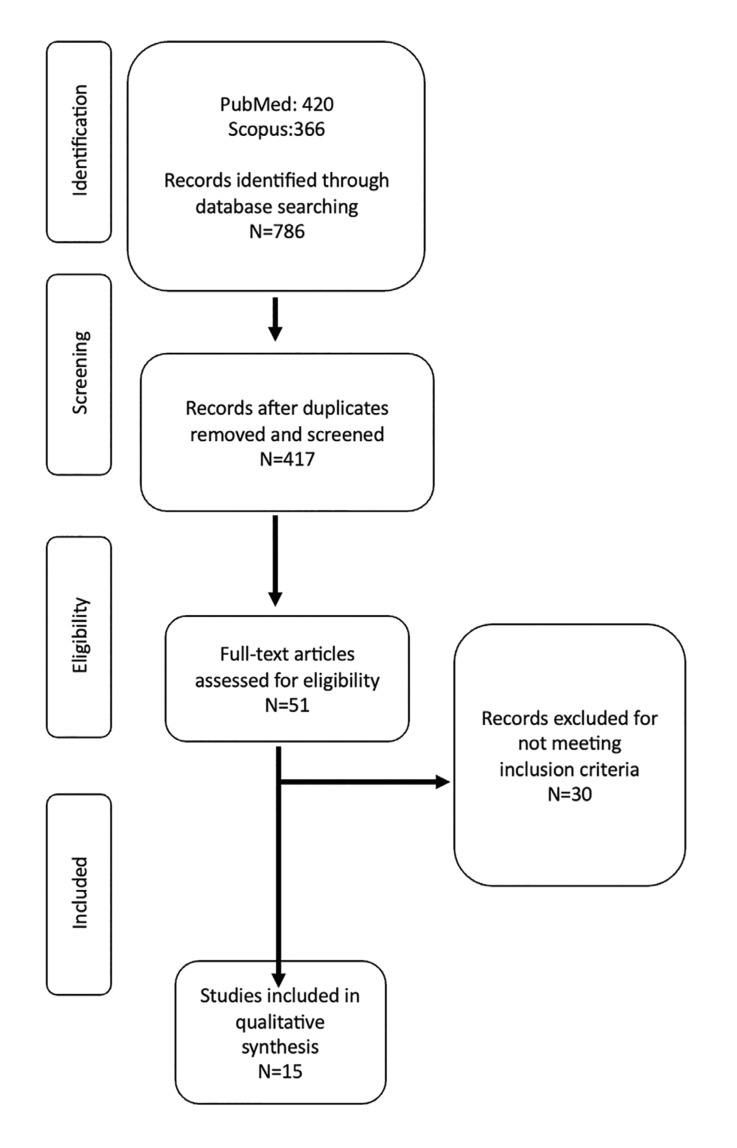
PRISMA® flow diagram of the search.

The search strategy produced 786 results (420 on PubMed, 366 on Scopus). After removing duplicates, three independent researchers (J.M., S.A., and JF. P-C.) reviewed the titles and abstracts of the articles and excluded 369 articles outside this review's scope and did not meet the inclusion criteria, resulting in 51 remaining articles. After a full-text review, 30 articles were excluded (Table 2) and 15 articles were eligible for inclusion in the current systematic review (Fig. 1) (14-28).

- Study Characteristics

In the present systematic review and meta-analysis, 12 included articles were prospective clinical studies, while 3 articles were randomized controlled clinical studies. Table 3 shows the characteristics of the included articles. Regarding the follow-up period, 8 studies included a follow-up period of 12 months, 3 studies included a follow-up period of 3 months, while the rest of the studies included a follow-up period of 54, 60, 72, and 120 months.

- Narrow versus Regular Diameter Implants

In the study by De Souza *et al*. 2018, 22 patients were recruited to evaluate the performance of narrow-diameter (3.3mm) and regular-diameter (4.1mm) implants supporting single crowns in the posterior regions of the jaws over three years. The study yielded prosthetic success rates of 90% for narrow implants and 95% for regular implants after three years, with issues primarily arising from chipping or screw loosening. No statistically significant differences were observed concerning marginal bone loss (14).

Benic *et al*.2013 compared 18 regular diameter 4.1 implants with 20 narrow diameter implants. The survival rate in both groups was 100% after a year of follow-up. The marginal bone level after one year did not show any significant difference between both groups, being 0.4 +- 0.53 mm (SD) in the 4.1 mm diameter and 0.41+- 0.66 mm in the 3.3 mm diameter implants (15).

Nilson *et al*. 2017 is a prospective study that included 52 patients and 69 implants, a total of 59 implants of 3.3 mm diameter and 10 implants with a 4.1 mm diameter. Implants showed a 100% survival after the follow-up (16).

- Survival and Success rate of Narrow Diameter implants

In a separate study conducted by De Souza *et al*. (2018), the authors found that prosthetic success rates of 90% were recorded for narrow implants and 95% for regular implants after three years (14).

Benic *et al*.2013 compared regular diameter implants with narrow diameter implants and found that the survival rate was 100% in both groups after a year of follow-up (15).

Nilson *et al*. 2017 showed 100% of survival after the follow-up in both regular diameter and narrow-diameter implants (16).

Gouveia *et al*. (2022) conducted a prospective clinical study where a total of 30 narrow-diameter implants, all with a 3.3mm diameter, were placed in 30 patients, utilizing two different implant surfaces. However, the surface type did not impact the implant survival rate. The study observed that the one-year follow-up survival rate was 100% (17).

In a randomized, controlled, multicenter clinical study, Ghazal *et al*. (2019), focused on single-tooth replacement in the anterior region with a sample size of 50 patients. A total of 30 narrow diameter implants, all with a 3.3 mm diameter, were placed in 30 patients, utilizing two different implant surfaces. However, the surface type did not impact implant survival or marginal bone loss. The study observed no implant losses (survival rate 100%) (18).

Kolinski *et al*. (2018) reported preliminary 1-year results from a prospective multicenter study involving 3.0-mm-diameter tapered implants. The study included 77 patients who received a total of 91 implants. After one year, 71 patients and 82 implants remained in the study, 3 implants failed, indicating a CSR of 96.7% (19).

Galindo-Moreno *et al*. (2017) conducted a 5-year follow-up study involving narrow-diameter implants in lateral incisors of the maxilla and central and lateral incisors in the mandible, focusing on early loading. A total of 69 patients received 97 implants, with four implants lost before loading (4.12% failure rate) (20).

Mangano *et al*. (2014) conducted a 10-year follow-up study with 279 patients and 324 3.3mm implants (Implant Brand Leone with Morse Taper Conical Connection). Four implants failed, resulting in a cumulative survival rate of 98.7% (21).

Xiao *et al*. (2020) carried out a 1-year prospective study evaluating the influence of implant location on titanium-zirconium alloy narrow-diameter. The study included 84 patients distributed across three groups: anterior, premolar, and molar. Interestingly, the anterior group had a 95.65% survival rate, while both premolar and molar groups achieved 100% (22).

Zinsli *et al*. (2004) reported on a 10-year prospective study involving 149 patients and 298 3.3mm implants. The study included a 10-year observation period, with restorations placed after 3-6 months. The cumulative 5-year survival rate for the implants was 98.7%, dropping slightly to 96.6% after 6 years (23).

Degidi *et al*. 2009 performed a prospective clinical study that included 60 patients with missing lateral incisors in the maxilla. Sixty implants were 3.0 mm diameter implants and had to be placed in healed sites. Patients were randomized into two treatment groups: 30 patients with 30 implants in the immediate restoration group and 30 patients with 30 implants in the one-stage group. All implants were sTable and osseointegrated at 6-month follow-up with a 100% implant survival rate in both groups (24).

Roccuzzo *et al*. 2022 performed a 1-year prospective clinical study to report radiographic, clinical, esthetic and patient-related outcomes on patients rehabilitated with narrow diameter implants with congenitally missing lateral incisors. The study included 100 patients who received 100 narrow-diameter dental implants, 2.9 mm (*n*=50) or 3.3 mm (*n*=50). At one year follow-up, 93 patients were available for evaluation. The study recorded loss of one 3.3 mm implant before loading (early implant failure) leading to an implant survival rate of 100% in the 2.9 mm group and 98% in the 3.3 mm group. No statistically significant difference was found between the two groups regarding implant survival (*P*=1.000; CI: 94.6%-99.9%) (25).

King *et al*. 2016 was a prospective, multicenter study that included 38 patients with maxillary lateral incisor hypodontia or mandibular incisor hypodontia treated with 62 small diameter 3.0 mm dental implants. The implant survival rate was 96.8% (two implants were lost during the healing period) (26).

Oyama *et al*. 2012 performed a 1-year prospective study with a sample size of 13 patients and 17 narrow diameter 3.0 mm dental implants to evaluate implant success rate and peri-implant tissue response at maxillary and mandibular incisor sites. Nine implants were placed on the site of the maxillary lateral incisor, while four implants were placed on the site of the mandibular central and lateral incisor. Implants were immediately provisionalized and parameters were evaluated immediately after implant placement and at 3,6 and 12 months after the surgery. At 12 months follow-up, the implant success rate was 100% (27).

- Clinical parameters evaluation of narrow diameter implants

When assessing clinical parameters related to narrow diameter implants, De Souza *et al*. (2018) showed no statistically significant differences were observed concerning marginal bone loss (14).

Benic *et al*. 2013 at a one-year follow-up of 38 patients (18 implants with a 4.1 mm diameter and 20 implants with a 3.3 diameter) demonstrated that marginal bone level did not show any significant difference between both groups, being of 0.4 +- 0.53 mm (SD) in the 4.1 mm diameter and 0.41+- 0.66 mm in the 3.3 mm diameter implants (15).

Gouveia *et al*. (2022) observed a mean marginal bone loss of -0.36 mm at the one-year follow-up. The findings suggest that narrow implants exhibit reduced marginal bone loss, maintaining sTable peri-implant tissues after one year (17).

Ghazal *et al*. (2019), assessed marginal bone loss, implant success, gingival recession, and patient satisfaction. The narrow diameter implant group exhibited lower mean bone loss (-0.27 mm) compared to the standard diameter group (0.48), demonstrating that both implant types were effective, with narrow implants having less marginal bone loss (18).

Kolinski *et al*. (2018) reported marginal bone loss was measured 0.57mm after 6 months and 0.25mm after 12 months. All implants used in the study were 3.0mm Nobel Active implants (19).

Galindo-Moreno *et al*. (2017) showed that marginal bone levels remained sTable during the first year (-0.11mm). After 5 years 8.43% experienced more than 1 mm of bone loss. The study utilized Dentsply Osseospeed 3.0mm implants (20).

Mangano *et al*. (2014) reported mean marginal bone loss at 10 years 0.69 mm (21).

Xiao *et al*. (2020) showed that marginal bone loss after 12 months showed no statistical significance between the three groups, with values of 0.68mm for anterior, 0.48mm for premolar, and 0.81mm for molar implants (22).

Degidi *et al*. 2009 measured mean marginal bone loss, probing depth, and bleeding on probing at 6, 12, 24, and 36-month follow-up visits. At the 36-month follow-up, mean marginal bone loss and probing depth for the immediate loading group were 0.85±0.71 mm and 1.91±0.59 mm respectively, and 0.75±0.63 mm and 2.27 ±0.81 mm for the one-stage group respectively. The authors found no statistically significant difference in the tested parameters between the two groups in the treatment of a single missing lateral incisor (24).

Roccuzzo *et al*. 2022 found that crestal bone loss of -0.19±0.25 mm was recorded for the 2.9 mm group, while for the 3.3 mm group was -0.25±0.31 mm with no statistically significant difference between the two groups (*P*=.342). Mean peri-implant probing depth was 2.55±0.41 mm in the 2.9 mm group and 2.50±0.45 mm in the 3.3 mm group with no statistically significant difference between the groups (*P*=.576) (25).

King *et al*. 2016 assessed bleeding on probing, gingival zenith score, and probing pocket depth after 6,12,24 and 36 months follow up while radiographic examination was assessed at 6,12 and 36 months follow-up visit. Mean marginal bone loss at follow-up visits at 6,12, and 36 months were 0.39 mm, 0.22 mm, and 0.23 mm respectively. No statistically significant differences were found regarding probing depth, bleeding on probing, and gingival zenith score (26).

Oyama *et al*. 2012 found that the mean marginal bone level after implant placement and 3,6 and 12 months was -0.03±0.06 mm, -0.28±0.35 mm, -0.28±0.32 mm, and -0.38±0.36 mm respectively. The mean marginal bone level change after implant placement and 3,6 and 12 months was -0.35±0.35 mm (27).

Knobloch *et al*. 2022 conducted a prospective cohort study with 14 patients who were diagnosed with cleft palate and congenitally missing lateral incisors and rehabilitated with 17 narrow diameter implants (3 mm and 3.5mm). The authors measured mean marginal bone loss and probe depth after 1 year of follow-up. The mean marginal bone loss at 1-year follow-up for all narrow diameter implants was 0.601±0.48mm. Regarding probing depth, the authors found that location (*P*=.012) and time (*P*=.009) were significant. A significant difference was found between the buccal and distal sites (*P*=.006) regarding baseline and 1-year follow-up (28).

- Complications

De Souza *et al*. 2018 evaluated the performance of narrow-diameter (3.3mm) and regular-diameter (4.1mm) implants supporting single crowns in the posterior regions of the jaws over three years. The study yielded prosthetic success rates of 90% for narrow implants and 95% for regular implants after three years, with issues primarily arising from chipping or screw loosening (14).

Zinsli *et al*. (2004) reported on a 10-year prospective study involving 149 patients and 298 3.3mm implants. The study included a 10-year observation period, with restorations placed after 3-6 months. Three implants were lost during healing, and two implants broke (one after 2 years and the other after 6 years). Both broken implants were 8mm long. Four implants exhibited transient peri-implantitis (23).

Roccuzzo *et al*. 2022 showed at a one-year follow-up with 93 patients who were available for evaluation, technical complications occurred once in three patients with no statistically significant difference between the groups(P>.05). Regarding OHIP scores, no significant differences were found between two groups (*P*=.110) (25).

Oyama *et al*. 2012 showed in their study that prosthetic complications occurred within the 3 months after surgery- seven fractured provisional restorations, two deboned provisional restorations three loosened abutment screws (27).

- Meta-analysis

**Figure 2:**
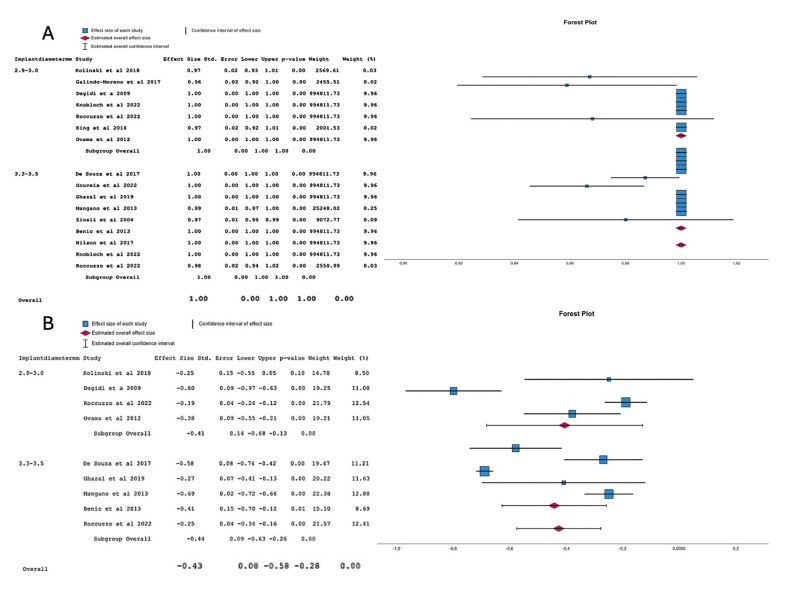
A Meta-analysis of the survival, B. Meta-analysis of the marginal bone loss variable.

Both groups demonstrated an implant survival rate of 99% (95% CI, 99.9%-100%), with no significant difference between them (*p*=0.933). A noTable 99% survival rate was observed for the narrow implants (2.9 to 3.5 mm) (95% CI, 99.9%-100%). This significant 99% survival rate was present in both the 2.9-3.0 mm and the 3.3-3.5 mm groups (95% CI, 99.9%-100%, *p*<0.001). Refer to Fig. 2.

There are no significant differences in the comparison between the 2.9-3.0 mm group and the 3.3-3.5 mm group (*p*=0.933). Both groups had a 99% survival (95% CI, 99.9%-100%). Refer to Fig. 2.

A significant marginal bone loss of -0.428 mm (95% CI: -0.576 to -0.279) was observed in both groups combined. There were no significant differences between the 2.9-3.0 mm group and the 3.3-3.5 mm group (*p*=0.828), with mean marginal bone losses of -0.407 mm (95% CI: -0.683 to -0.131) and -0.444 mm (95% CI: -0.628 to -0.259), respectively. Refer to Fig. 2 for more details.

**Figure 3:**
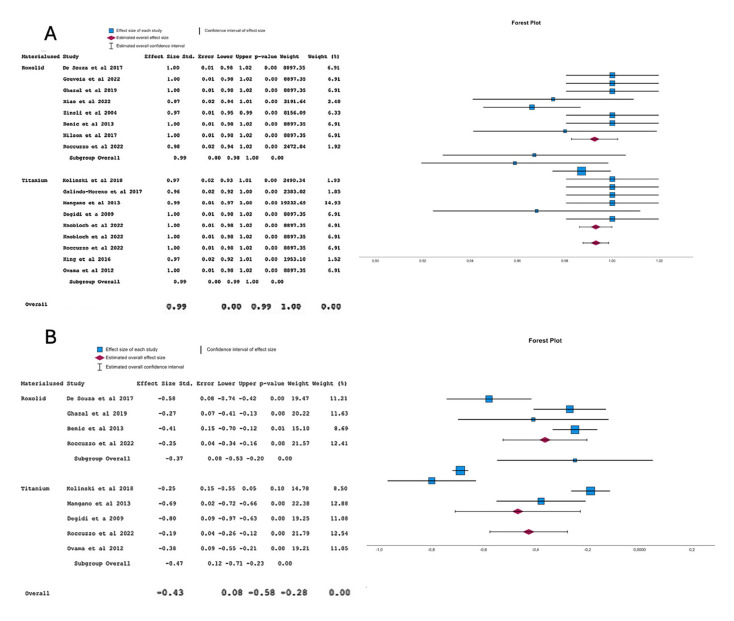
A.Meta-analysis of the survival between titanium-zirconia and titanium implants, B. Meta-analysis of the margin:al bone loss variable in titanium-zirconia and titanium implants.

A significant 99% survival of the titanium-zirconia and titanium implants was registered (95% CI, 99%-100%). No significant differences exist in the comparison between the titanium-zirconia and titanium implants. Both groups had a 99% survival (95% CI, 99%-100%). Refer to Fig. 3.

A significant marginal bone loss of -0,43 mm (CI95%: -0,58 to -0,28) was registered in both groups together (titanium-zirconia and titanium implants). Titanium-zirconia implants presented a mean of marginal bone loss of -0,37 mm (CI, 95%: -0,53 to -0,20) meanwhile, titanium implants had a mean of marginal bone loss of -0,43 mm (CI95%:-0,71 to -0,28). Refer to Fig. 3.

**Figure 4:**
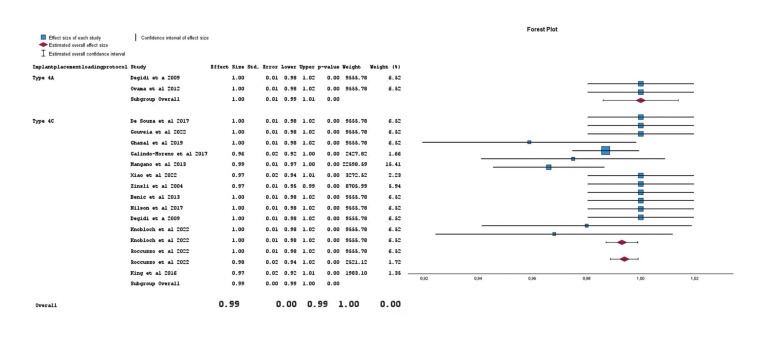
Meta-analysis of the survival variable according to the implant placement and loading protocol.

Most of the articles included cases with delayed implant placement and delayed loading (Type 4C protocol according to Gallucci *et al*. 2018) (29). Only two articles (24,27) included cases with immediate loading after delayed implant placement (Type 4A protocol according to Gallucci *et al*. 2018) (29). When comparing Type 4A vs Type 4C in narrow implants, the meta-analysis did not find differences regarding implant bone loss or implant survival. Refer to Fig. 4.

## Discussion

The current systematic review aimed to evaluate the predictability of narrow-diameter implants as a treatment alternative in clinical situations characterized by restricted mesiodistal space or limited bone availability, where using regular diameter implants might need more complex procedures such as guided bone regeneration. Additionally, the study sought to investigate potential significant differences between two subcategories of narrow-diameter implants.

For this systematic review, narrow-diameter implants were divided into groups according to their diameter, with a 2.9-3.3mm group and a 3.3-3.5mm group, but they were also divided into subgroups according to their material, with a group for pure titanium implants and a group for titanium-zirconia alloys.

The included studies comparing regular and narrow-diameter implants showed no statistically significant differences regarding implant survival rates, success rates, and marginal bone loss. Studies included in the current review showed a high survival rate for narrow-diameter implants ranging between 95.65 and 100 %. Previous studies on regular diameter implants reported a survival rate of 97.2% after 5 years (30). The mean marginal bone loss for narrow diameter implants was -0,428 mm (CI95%: -0,576 to -0,279) in the included studies. These results are comparable with the reported mean marginal bone loss of -1.25 mm to -0.10mm around regular-diameter implants (31).

The findings of our study align with existing literature affirming the high survival and success rates associated with narrow implants. These results are consistent with prior research, such as Chiapasco *et al*. (32) who reported a TI-Zr implant survival rate of 100% within a range of 3-19 months. Altuna *et al*. (33) observed marginal bone loss mean values of 0.36 ± 0.06mm after 1 year and 0.41 ± 0.09mm after 2 years, and survival rates of 98.4% and 97.8% at 1-year post-implant placement, closely resembling our review outcomes. Quirynen *et al*. (34) also corroborate our findings even using multiple implant placements in the mandible, noting comparable outcomes between titanium and titanium-zirconia implants. Their study revealed survival rates of 98.7% and 97.3% and marginal bone level changes of -0.78 ± 0.75mm and -0.60 ± 0.71mm for titanium-zirconia and titanium implants, respectively. Importantly, no significant differences were observed between these two implant types in terms of bone-level change, soft tissue parameters, or overall survival and success rates, further supporting the results drawn from our review.

Based on the comparable survival rates and marginal bone loss observed for narrow and regular diameter implants, the use of narrow diameter implants might be a predicTable treatment option in clinical scenarios characterized by limited space or insufficient bone for regular diameter implants, where the placement of regular diameter implants would be only feasible with extended procedures such as additional bone grafting measures.

Comparing two subgroups of narrow-diameter implants, the results of the meta-analysis showed no significant differences between the 2.9-3.3mm group and the 3.3-3.5mm group regarding implant survival and marginal bone loss. These results are comparable to the previous consensus statement showing that only narrow-diameter implants with an implant diameter of less than 2.5 mm resulted in significantly different survival rates (8).

The Included studies exhibited a wide range of follow-up periods, and narrow implants within these studies varied in terms of implant lengths, implant surfaces, and materials used. These differences posed a limitation to the comparability of the study results.

Furthermore, technical complication rates were insufficiently described in the included studies. Complications were heterogeneously registered, and definitions of success criteria were lacking. A more standardized approach to defining success criteria and registering complication rates is recommended for future studies.

## Conclusions

The findings reveal no significant differences between 2.9-3.3mm and 3.3-3.5mm groups within narrow implants. Both titanium and titanium-zirconia alloys in narrow implants, exhibit comparable high survival rates and minimal marginal bone loss, showcasing their feasibility as a viable treatment alternative in scenarios with limited space or inadequate bone for regular diameter implants.

## Figures and Tables

**Table 1:** Risk bias assessment Newcastle-Ottawa Scale.

Study	Study	De Souza *et al.* 2017	Gouveia *et al.* 2022	Ghazal *et al.* 2019	Kolinski *et al.* 2018	Galindo-Moreno *et al.* 2017	Mangano *et al.* 2013	Xiao *et al.* 2022	Zinsli *et al.* 2004	Benic *et al.* 2013	Nilson *et al.* 2017	Degidi et a 2009	Knobloch *et al.* 2022	Roccuzzo *et al.* 2022	King *et al.* 2016	Ovama *et al.* 2012
Selection	Representivenessof the exposed cohort（1）	*	*	*	*	*	*	*	*	*	*	*	*	*	*	*
Selection	Selection of thenon-exposed cohort（1）	*	*	*	*	*	*	*	*	*	*	*	*	*	*	*
Selection	Ascertainment of exposure（1）	*	*	*	*	-	*	-	-	*	-	*	*	*	*	*
Selection	Demonstration that outcome of interest was not present at start ofstudy（1）	*	*	*	*	*	*	*	*	*	*	*	*	*	*	*
Comparability	Compare ability of cohorts on the basis of the design or analysis（2）	**	**	**	**	**	**	**	**	**	**	**	**	**	**	**
Outcome	Assessment of outcome（1）	*	*	*	*	-	*	-	-	*	-	*	*	*	*	*
Outcome	Was follow up long enough for outcomes tooccur（1）	*	*	*	*	*	*	*	*	*	*	*	*	*	*	*
Outcome	Adequacy offollow up of cohorts（1)	*	*	*	*	*	*	*	*	*	*	*	*	*	*	*
Total score		Good quality	Good quality	Good quality	Good quality	Medium quality	Good quality	Medium quality	Medium quality	Good quality	Medium quality	Good quality	Good quality	Good quality	Good quality	Good quality

**Table 2:** Full-text articles reviewed and excluded, with specific exclusion criteria.

Reason for exclusion criteria	Author, year
Multiple narrow implant rehabilitations included on the sample	Bielemann *et al.*. (2022)
Multiple narrow implant rehabilitations included on the sample	Threeburuth *et al.*. (2018)
Multiple narrow implant rehabilitations included on the sample	Reis *et al.*. (2019)
Multiple narrow implant rehabilitations included on the sample	Bielemann *et al.*. (2018),
Multiple narrow implant rehabilitations included on the sample	Mundt *et al.*. (2023)
Multiple narrow implant rehabilitations included on the sample	Enkling *et al.*. (2020)
Multiple narrow implant rehabilitations included on the sample	Hallman *et al.*. (2001)
Multiple narrow implant rehabilitations included on the sample	Tolentino *et al.*. (2013)
Multiple narrow implant rehabilitations included on the sample	Temizel *et al.*. (2017)
Multiple narrow implant rehabilitations included on the sample	Enkling *et al.*. (2017)
Multiple narrow implant rehabilitations included on the sample	Moráguez *et al.*. (2017)
Multiple narrow implant rehabilitations included on the sample	Worni *et al.*. (2018)
Multiple narrow implant rehabilitations included on the sample	Antiua *et al.*. (2022)
Multiple narrow implant rehabilitations included on the sample	Van Doorne *et al.*. (2023)
Multiple narrow implant rehabilitations included on the sample	Altuna *et al.*. (2023)
Multiple narrow implant rehabilitations included on the sample	Maryod *et al.*. (2014)
Multiple narrow implant rehabilitations included on the sample	Curado *et al.*. (2023)
Multiple narrow implant rehabilitations included on the sample	Müller *et al.*. (2015)
Multiple narrow implant rehabilitations included on the sample	Al-Nawas *et al.*. (2012)
Multiple narrow implant rehabilitations included on the sample and less than 12 months of follow-up	Leles *et al.*. (2022)
Multiple narrow implant rehabilitations included on the sample	Giannakopoulos *et al.*. (2012)
Multiple narrow implant rehabilitations included on the sample	de Souza *et al.*. (2015)
It does not register survival	Rocuzzo *et al.*. (2021)
Include majority of patients under 18 years old. Mean age of 12 years old	Ferreira *et al.*. (2023)
Less than 12 months of follow-up	Cabrera-Dominguez *et al.*. 2017

**Table 3:** Characteristics of the included articles.

Reference	De Souza *et al.* 2017	Gouveia *et al.* 2022	Ghazal *et al.* 2019	Kolinski *et al.* 2018	Galindo-Moreno *et al.* 2017	Mangano *et al.* 2013	Xiao *et al.* 2022	Zinsli *et al.* 2004	Benic *et al.* 2013	Nilson *et al.* 2017	Degidi et a 2009	Knobloch *et al.* 2022	Roccuzzo *et al.* 2022	King *et al.* 2016	Ovama *et al.* 2012
Number of patients	22	30	47	71	69	279	80	149	38	52	60	14	100	38	13
Number of implants	44 (22 3.3mm - 22 4.1mm)	30	47 (23 3.3mm - 24 4.1mm)	82	97	324	81	298	38 (20 3.3mm - 18 4.1mm)	69 (59 3.3mm - 10 4.1mm)	60	17	100 (only 93 analyzed)	62	17
Follow-up (months)	36	12	12	12	60	120	12	72	12	54	36	12	12	36	12
Survival %	100	100	100	96.7	95.88	98.7	95.65% Anterior100 %Premolar100% Molar	96.6	100 (both groups)	100	100	100	100% for 2.9 and 98% for 3.3	96.8	100
Implant failures	0	0	0	3	4	4	2	5	0	0	0	0	1 (3.3mm)	2	0
Complications	10% prosthetic failures after 3 years (chipping and screw loosening)	1 patient with mucositis	None	Postoperative pain, fracture of temporary crown, occlusal Trauma, abutment screw fractures	Crowns lost and abutments fractures (17)	3 patients with swelling, 1 patient with periimplantitis (4yrs later), 7 patients had loss of retention, 3 patients had porcelain fractures	None described	Fractures and Peri-Implantitis (infections)	None	Zirconia Abutment fracture in 12.5% of implants	Bone loss for 1 patient, 1 patient had provisional prosthesis fracture, 1 patient had Screw loosening	None	Technical complications	5 patients had abutments fractures and 1 patient had screw abutment loose. Eight crowns lost their retention, one patient had swelling, 1 patient excess cement	Seven patients fractured the provisional restorations, two patients had a debonded provisional, Three patients loosened abutment screws
Implant length	6 mm 8 mm 10 mm 12 mm	10 mm 12 mm 14 mm	Not specified	10 mm 11,5 mm 13 mm 15 mm	11 mm 13 mm 15 mm	8mm10 mm12 mm14 mm	8 - 12mm	8 mm 10 mm 12 mm	8 mm 10 mm 12 mm 14 mm	10 mm 12 mm	13 mm 15 mm	11 mm 13 mm	10 mm 12 mm	11 mm 13 mm 15 mm	At least 11 but not specified
Implant diameter (mm)	3.3	3.3	3.3	3	3	3.3	3.3	3.3	3.3	3.3	3.0	3.0 and 3.5	2.9 and 3.3	3.0	3.0
Implant material	Titanium- zirconia	Titanium- zirconia	Titanium- zirconia	Titanium	Titanium	Titanium	Titanium- zirconia	Titanium- zirconia	Titanium- zirconia	Titanium- zirconia	Titanium	Titanium	Titanium- zirconia	Titanium	Titanium
Implant placement timing type	Delayed implant placement	Delayed placement	Delayed placement	Immediate and delayed placement	Delayed Placement	Delayed Placement	Delayed Placement	Delayed Placement	Delayed Placement	Delayed Placement	Delayed placement	Delayed Placement	Delayed placement	Delayed placement	Delayed placement
Loading type	Delayed implant loading	Delayed loading	Delayed loading	Immediately loaded	Delayed Loading	Delayed Loading	Delayed Loading	Delayed Loading	Delayed Loading	Delayed Loading	Immediate load vs. delayed	Delayed Loading	Delayed loading	Delayed loading	Immediate load
Placement/Loading protocol	Type 4C	Type 4C	Type 4C	No specified each case	Type 4C	Type 4C	Type 4C	Type 4C	Type 4C	Type 4C	Type 4A 30 implantsType 4C 30 implants	Type 4C	Type 4C	Type 4C	Type 4A
Tooth replaced	Premolar and Molar position (UJ and LJ)	Tooth bounded gaps	Anterior and premolar	Maxillary lat. incisor Mandibular lat. and central incisor	12, 22, 32, 31, 41 or 42	38.6% wereincisors, 36.7% were premolars, 13.0% werecanines, and 11.7% were molars	Anterior, Premolar, Molar	All regions were included	Anterior and premolar	Anterior	Maxillary lateral incisor	Maxillary lateral incisor	Maxillary lateral incisor	Maxillary lateral incisor	Anterior (UJ/LJ)
GBR associated	No	Yes (15 Patients)	No	Yes (22%)	Some implants had autologous chips grafted around the neck	No	No	No	50% of cases had simultaneous GBR	19 patients with GBR (4 at time of implant placement)	No	2 received bone graft before implant placement	In case of fenestration or dehiscence type defects	covering of themost coronal threads of the implants with autogenousbone chips was allowed	No
Marginal Bone Loss (mm)	-0.58 ± 0.39 mm	-0.36 mm (0 mm to - 1.77 mm)	-0.27 ± 0.34 mm	− 0.25 ± 1.38 mm	-	0.69 ± 0.28 mm	-	-	−0.41 ± 0.66 mm	-	-0.85± 0.71 mm-0.75± 0.63 mm	-0.601 ±0.48 mm	-0.19± 0.25 mm (2.9 implant diameter) -0.25± 0.31 (3.3 implant diameter)	0.23mm	-0.38 ± 0.36 mm
